# ID 52 - Boletim do Instituto de Saúde (BIS) – Participação Social na Avaliação de Tecnologias em Saúde: acesso e democracia

**DOI:** 10.5327/2237-9622.2025.v34s1.52

**Published:** 2025-11-25

**Authors:** Fotini Santos Toscas, Andrea Brígida de Souza, Marília Mastrocolla de Almeida Cardoso, Marisa da Silva Santos

## Abstract

**Introdução:**

O Boletim do Instituto de Saúde (BIS) é uma publicação semestral dedicada a promover debates com foco na melhoria do Sistema Único de Saúde (SUS). A edição temática "Participação social na Avaliação de Tecnologias em Saúde: acesso e democracia" tem como objetivo envolver os leitores na compreensão de como a sociedade pode contribuir no processo de incorporação de tecnologias no SUS, promovendo maior transparência, equidade e confiança nas decisões de saúde.

A edição conta com a participação de 40 autores e coautores, totalizando 16 trabalhos, que abordam a importância da participação social no processo de Avaliação de Tecnologias em Saúde (ATS). Entendendo que a participação ativa da sociedade é essencial para garantir o acesso equitativo a novas tecnologias e reduzir desigualdades no SUS.

Os trabalhos iluminam o protagonismo das associações de pacientes, do controle social e dos movimentos associativos. Aprofundam o debate da importância e limitações das ferramentas de consulta pública. Trazem à tona a relevância da avaliação da evidência qualitativa e dos mecanismos de participação direta, com os relatos de experiências.

As alterações no processo de ATS e incorporação de tecnologias oncológicas, decorrentes da recente Lei da Política Nacional de Prevenção e Controle do Câncer, são examinadas e discutidas com estudo de caso, oferecendo uma visão abrangente do panorama atual. As reversões das recomendações são abordadas no contexto das doenças raras e no âmbito da Política Nacional de Atenção Integral às Pessoas com Doenças Raras.

A edição também apresenta ensaios que incentivam reflexões sobre bioética, direitos humanos dos pacientes e os desafios relacionados à equidade no acesso a novas tecnologias. Relatos de experiências ilustram a participação social na Conitec e em conferências nacionais de saúde, destacando a importância do engajamento público no fortalecimento da democracia participativa.

Os relatos de experiência oferecem percepções sobre a participação social na Comissão Nacional de Incorporação de Tecnologias no Sistema Único de Saúde (Conitec), incluindo atividades em conferências nacionais de saúde. São apresentados casos específicos que ilustram o envolvimento público para o pleno exercício da cidadania e a materialização da democracia participativa.

Uma entrevista com Vania Canuto, intitulada "Uma história com nome de gente", narra a evolução dos mecanismos de participação social nos 13 anos da Conitec. A entrevista oferece uma perspectiva sobre o papel da sociedade na construção do SUS e na ATS, além de apresentar um panorama futuro da participação social no contexto das Américas.

**Método:**

Pretende-se, durante o V Congresso da Rebrats, divulgar a edição apresentando brevemente os trabalhos, estimular a produção científica e provocar discussão sobre a participação social na ATS. A dinâmica busca explorar os textos como forma de uma jornada envolvente pelo universo da democratização e ampla participação social na ATS. Será um espaço oportuno para compartilhar como a participação ativa dos cidadãos pode moldar políticas públicas de saúde, garantir acesso equitativo a tecnologias, divulgar relatos inspiradores e experiências práticas de engajamento e participação social.

Para tanto, será necessário um ambiente equipado com sistema de som e projeção, proporcionando um espaço para divulgação de experiências sobre como a participação cidadã pode influenciar políticas públicas e garantir acesso equitativo às tecnologias de saúde.

